# MDFE-Net: a multiscale dilated feature enhancement network for small object detection

**DOI:** 10.3389/fpls.2026.1778795

**Published:** 2026-02-24

**Authors:** Tianzhe Liu, Shihang Lin, Jiayi Zhang, Bin Li, Junyan Zhu

**Affiliations:** 1 Fujian Police College, Fuzhou, China; 2 College of Computer and Information Science, Fujian Agriculture and Forestry University, Fuzhou, Fujian, China

**Keywords:** context feature, dilated convolution, feature enhancement, multiscale, small object detection

## Abstract

Due to the lack of feature information and complex background, the task of small object detection is very challenging. To solve these problems, this paper proposes two small object detection performance enhancement modules for multiple detection tasks and an efficient small object detection network called Multiscale Dilate Feature Enhancement Network (MDFE-Net). MDFE-Net includes two innovative plug-and-play modules: the multi-scale dilated feature aggregation (MDFA) module and the context feature enhancement (CFE) module. MDFA improves the efficiency of multi-scale feature fusion, which is used to capture multi-scale context information and improve the expression of underlying feature information. CFE improves the local feature perception and preserves and extracts the effective information of small image objects to the maximum extent. The network enhances the perception of small objects' feature information and restrains the problem of complex and confusing backgrounds to some extent. We used two public datasets (VisDrone and GTSDB) and a self-built agricultural small object dataset (PSD-Node) to verify the effectiveness of the method. On the above three datasets, the AP50 of MDFE-Net reached 0.304, 0.952, and 0.895, and the AP is 0.172, 0.805, and 0.476, respectively, which exceeded the benchmark model and the current SOTA method.This research presents an innovative small object detection network and provides a reliable technical solution for agricultural small object detection.

## 1 Introduction

In object detection, small-object detection is an important yet challenging task. In recent years, due to the rapid development and exploration of remote sensing technology (Tong et al., 2020; Shimoni et al., 2019) in the fields of UAV aerial photography, traffic monitoring, and smart agriculture, the research on small object detection has made remarkable progress. However, small target detection has two main difficulties: (1) limited feature representation caused by small object size and low pixel count, and (2) frequent occlusion and confusion arising from complex image backgrounds (Ruan et al., 2023), which leads to additional difficulties in the detection of small objects by models. Therefore, small object detection has always been one of the most challenging tasks in object detection (Yang et al., 2015).

Recently, numerous studies have contributed significantly to addressing small object detection. Kisantal et al (Kisantal et al., 2019). fully analyzed the principle of small object detection and proposed an oversampling image method to improve the small object detection performance of the model. Yuan et al (Yuan and Zhang, 2021). introduced an improved coupled network to solve the localization problem of small object detection. Zhang et al (Zhang et al., 2022). designed an adaptive dense pyramid network, which achieved excellent performance in dense small object detection tasks.

Motivated by the aforementioned challenges, we propose two novel plug-and-play modules specifically designed for improving small object detection across diverse scenarios. The key to alleviating the problem of insufficient feature information and background confusion lies in feature fusion and feature enhancement. For feature fusion, we combine multi-scale features with dilated convolution to make full use of multi-scale context information to effectively enhance the network’s perception of small objects. MDFA module is proposed to enrich the context features, increase the model’s receptive field, and promote the aggregation of more abundant underlying feature information so that the network can extract comprehensive information. The detection performance of small objects is improved. In terms of feature enhancement, we use a multi-branch convolution structure to extract richer semantic information and introduce dilated convolution to obtain richer local context information while expanding the receptive field. We propose a CFE module to enhance small object features in the process of network propagation by enhancing feature saturation and expanding the receptive field.

In this paper, we propose MDFE-Net, a multi-scale dilated feature enhancement network that incorporates two plug-and-play modules to improve small-object detection. The main contributions are summarized as follows:

1. We propose the MDFA module to enrich low-level feature representations by capturing multi-scale contextual information, alleviating the limited feature cues caused by small object sizes and few pixels. The module can be integrated into detection heads to enhance detection capability.
2. We design the CFE module to mitigate occlusion and background confusion by retaining and extracting informative cues for small objects through receptive-field expansion and multi-branch feature aggregation. The module can be embedded along the feature path from the backbone to the neck to strengthen feature information capture.
3. By integrating MDFA and CFE into YOLO11N, we build MDFE-Net and evaluate it on two public datasets (VisDrone and GTSDB) and one self-built dataset (PSD-Node). The experimental results show consistent improvements over strong baselines and competitive performance compared with state-of-the-art methods.

The rest of this paper is organized as follows: in the Section 2, the related work of small objects detection is introduced. Section 3 details the proposed MDFA and CFE modules and describes the architecture of MDFE-Net. Section 4 provides comprehensive experimental setups, comparative evaluations, and ablation studies demonstrating module effectiveness. Section 5 is the conclusion of this paper.

## 2 Related work

### 2.1 Small object detection

The definition of small objects is usually divided into two categories: relative size and absolute size. The relative size emphasizes the relationship between the object and the image size and generally emphasizes the proportion of the object to the image area. Chen et al (Girshick et al., 2014). made a specific definition of the small object through research: in the same category, the median ratio of the border area of the small object to the entire image area should be between 0.08% and 0.58%. The definition of the absolute size of a small object focuses on the pixel value of the object itself. Among them, the COCO dataset proposes that the pixels of small objects should be less than 32×32 pixels (Lin et al., 2014). In addition, different public datasets have different definitions of the absolute size of small objects, such as the WiderFace dataset (Yang et al., 2016), which defines a small object with a pixel range of 10 to 50, and the TinyPerson dataset (Yu et al., 2020), which defines a small object with a pixel range of 20 to 32. Recent works have proposed specialized designs for dense small-object detection. FBRT-YOLO (Xiao et al., 2025) introduces task-oriented improvements to enhance detection robustness in crowded scenes by strengthening feature representation and detection strategies for small targets. EDSOD (Li et al., 2025) presents a dedicated small-object detector that improves feature extraction and localization quality under challenging backgrounds, demonstrating competitive performance on public benchmarks. Small objects typically occupy only a few pixels in an image, which limits available visual cues and makes small-object detection one of the most challenging tasks in object detection.

### 2.2 Multi-scale feature fusion

Multi-scale means that in the process of deep learning, images or features with different resolutions are input, and different resolutions represent different scales. In object detection tasks, objects often appear in various sizes, and it is difficult to capture the features of all objects effectively with a single scale feature extraction. Therefore, multi-scale methods can better detect objects of different sizes by extracting features at different scales. Feature pyramid is a structure that deals with multi-scale feature information. By using many scale features, the network can extract more comprehensive information, so as to improve the detection effect of the network model on small objects. Feature Pyramid Network (Lin et al., 2017), as an enhanced feature architecture, is proposed to improve multi-scale problems well, and the performance of network models can be well improved by adding top-down paths to integrate multi-scale features. Feature pyramid-based object detection methods and many applied research methods have also achieved remarkable results in subsequent visual tasks. On the basis of the feature pyramid, PANet (Liu et al., 2018b) further improves the positioning capability of the feature pyramid by adding additional bottom-up feature paths, which can shorten the information path from the low layer to the high layer to enhance the feature hierarchy. EfficientDet (Tan et al., 2020) proposed a weighted bidirectional feature pyramid network (BiFPN), which introduced learnable weights for different input features in the fusion process, and a composite feature pyramid network scaling method. ASFF (Liu et al., 2019) research shows that small objects are usually associated with lower-level feature layers, while large objects are usually associated with higher-level feature layers. An adaptive spatial feature fusion method is proposed to promote the fusion of feature information by learning the correlation between different feature maps. SCRDet (Yang et al., 2019) designed a sampling fusion network, which fused multi-layer features with anchor sampling to improve the detection performance of the network model for small objects.

### 2.3 Feature enhancement

In object detection, the semantic expression of the model can be further enhanced by feature enhancement before the feature fusion. In this process, the feature expression and discrimination ability of the feature are improved through the fine processing of feature maps of different scales, so as to provide more abundant and accurate information for the subsequent feature fusion. Feature enhancement can be achieved through the attention mechanism: Cheng et al (Cheng et al., 2021). enhanced features by using the dual attention mechanism before fusion, which enhanced the sensitivity of the network to different features and enhanced the network detection performance. Zhang and Shen (Zhang and Shen, 2022) combined spatial attention mechanism and channel attention mechanism to form a feature enhancement module to enhance the features of the network. In addition to the attention mechanism, there are also two common feature enhancement methods: multi-branch convolution and transformer encoders (Liu et al., 2022; Li et al., 2023b).

## 3 Methods

### 3.1 Overview

This section presents two plug-and-play modules and the overall architecture of MDFE-Net. Specifically, the MDFA module captures multi-scale contextual information, and the CFE module enhances feature representation for small objects. We integrate these two modules together with a P2-level extra detect head (EDH) into the lightweight YOLO11N baseline, resulting in the proposed MDFE-Net.

### 3.2 MDFA module

**Figure 1:**
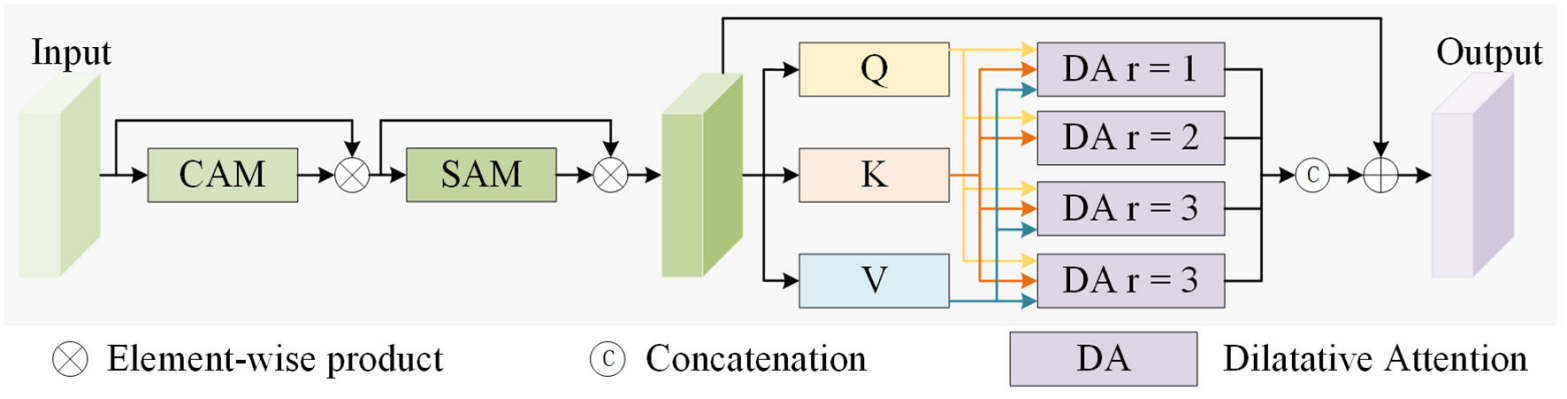
Overall structure of the MDFA module.

In object detection, hierarchical features are responsible for detecting the object of the corresponding size, and small objects are often small in size and lack sufficient features, which is difficult to accurately locate and detect. These problems will affect the accuracy and robustness of its detection. Low-level features contain rich location and local details. Effective use of low-level feature information can improve the localization and detection ability of smaller objects. In view of these problems, recent studies show that effective use of low-level features can significantly improve the detection ability of small objects. Low-level features (such as shallow convolution features) contain abundant location information and local details, which are of great significance for the accurate localization of small objects. However, relying only on low-level features may lack sufficient contextual information and global awareness, so a mechanism needs to be designed to combine low-level features with high-level semantic features to achieve comprehensive capture of multi-scale objects. Inspired by Dilate Former (Jiao et al., 2023), we proposed a new multi-scale dilated feature aggregation (MDFA) module, as shown in **Figure 1**, designed to effectively capture multi-scale context and enhance small-object detection performance.

**Figure 2:**
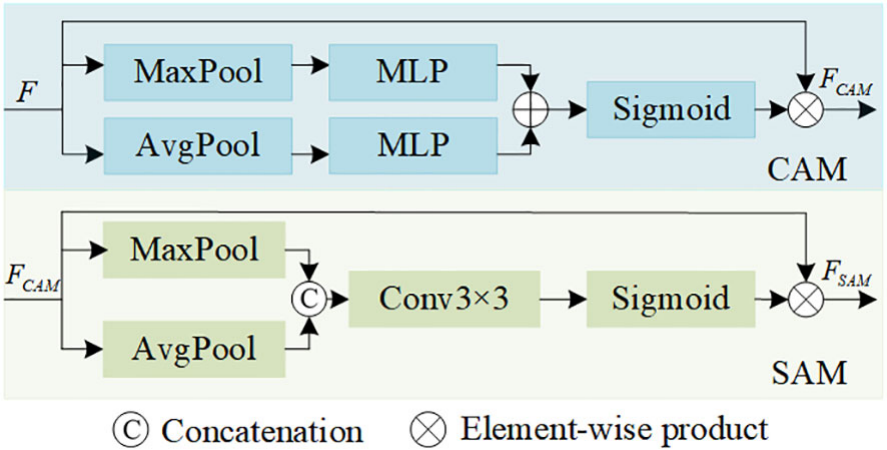
Overall structure of the CAM module and SAM module.

MDFA module aims to enhance the model’s ability to extract small object features by integrating the advantages of attention mechanism and dilated convolution while capturing rich context information in a multi-scale range. This module is divided into two main parts: convolutional block attention module and multi-scale dilated attention module (Woo et al., 2018), which work together to improve feature expressiveness and multi-scale adaptability. Firstly, **Figure 2** shows the channel attention module and the spatial attention module used in the previous part of the network structure. The channel attention mechanism highlights the more significant feature channels by weighted aggregation on the dimension of feature channels, thus improving the ability of the network to pay attention to important features. Accordingly, the spatial attention mechanism enhances the feature representation of key regions by applying attention weight in the spatial dimension. These two attention mechanisms are combined to form a convolutional block attention module to fully mine local details in features. The work flow of the convolutional block attention module is as follows: Input features are first processed by channel attention and spatial attention to generate two sets of attention weights; Then, by multiplying element by element, the attention weight is combined with the input features to obtain the enhanced feature representation. This process can effectively improve the sensitivity and representation ability of the network when dealing with small objects, especially in the complex background to better capture the key information of small objects. Secondly, we design a multi-scale dilated attention module inspired by the multi-head attention mechanism and dilated convolution. The core idea is to combine the characteristics of multi-head attention mechanism and dilated convolution to capture multi-scale context information. Specifically, the module first maps the features of the output of the convolution attention module to the query (Q), key (K), and value (V) spaces by linear projection, based on the principle of multi-head attention mechanism. Then, in order to enhance the expressiveness of features at different scales, we divide the feature channels into four groups and input them into four Dilated Attention head with dilated convolution with different dilated rates (r=1,2,3,4 respectively). Each Dilated Attention heads uses dilated convolution to expand the properties of the receptive field without increasing parameters, extracting features from different scales and context ranges. After processing with a Dilated Attention head, all feature outputs are fused through multi-scale aggregation operations to integrate information from multi-scale contexts.

However, relying solely on dilated convolution may have the following problems: On the one hand, the sparse sampling characteristics of dilated convolution may cause some fine-grained features to be ignored; On the other hand, a large dilated rate may result in an uneven distribution of receptive fields, resulting in insufficient attention to the characteristics of some regions. To address these issues, we designed a skip connection to introduce raw features into the aggregation process, further complementing fine-grained information and global consistency. This design not only avoids the loss of feature information but also improves the utilization efficiency of the original feature. The dilated attention mechanism enlarges the receptive field through dilated convolution, thereby demonstrating a greater ability to capture contextual information. In addition, compared with the traditional downsampling operation, this mechanism can retain more spatial details without reducing the resolution of the input image, which makes the model perform better in small object detection tasks. By combining the flexibility of the multi-head attention mechanism and the multi-scale characteristics of dilated convolution, the MDFA module achieves the efficient capture and utilization of multi-scale features, greatly improving the detection accuracy and robustness of the model when dealing with small objects. The formulas processed by the MDFA module are expressed as follows: Equations 1–4:

(1) $$W_{1}=f_{SAM}\left[f_{CAM}\left(F\right)⊗F]⊗[f_{CAM}\left(F\right)⊗F\right]$$

(2) $$K,Q,V=Linear\left(W_{1}\right)$$

(3) $$H_{i}=DA\left(Q_{i},K_{i},V_{i},r_{i}\right),1\leq i\leq n$$

(4) $$W_{out}=Linear\left(Concat\left(H_{1},\ldots ,H_{n}\right)\right)⊕W_{1}$$

Where *f_CAM_*(.) and *f_SAM_*(.) represent the input CAM and SAM modules for calculation operations, *DA*(.) represents dilated attention mechanism operates, *Linear* represent feature linear mapping, *Concat* represents feature mapping concatenation operation, ⊗ represents Element-wise Multiplication, ⊕ represents Element-wise Addition, *F* is the input feature, *W*_1_ is the output feature map that obtained after CAM and SAM module operation, *K, Q, V* represent value obtained after a linear mapping operation, *H_i_* is the output features that obtained after an dilated attention mechanism with an expansion rate of *i*, *W_out_* is the output feature.

### 3.3 CFE module

**Figure 3:**
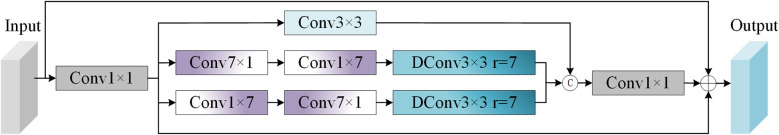
Overall structure of the CFE module.

Small objects are usually made up of only a few pixels in an image. The current mainstream object detection network typically consists of a backbone network, a neck network, and detect head three parts. The backbone network performs better for the detection of medium and large objects, but its feature extraction ability is limited for the detected objects with simple textures and small size. In the process of feature extraction, the features extracted by the backbone network often contain less semantic information and are limited by a narrow receptive field, which makes it difficult to distinguish the features of small objects from the occltors in the background, thus affecting the detection accuracy. To solve this problem, inspired by RFB-s (Liu et al., 2018a) and FFCA-YOLO (Zhang et al., 2024), we propose context feature enhancement (CFE) module, as shown in **Figure 3**.

CFE module enhances the expression ability of object features from two aspects: First, it enhances the feature saturation by adopting a multi-branch convolution structure to extract richer semantic information, so as to improve the feature expression ability of small objects. Second, it expands the receptive field of features to obtain more adequate local context information by introducing dilated convolution, so as to enhance the context awareness of small objects. This design can not only significantly improve the feature expression ability of small objects, but also optimize the computational efficiency and reduce the parameters of the network to a certain extent.

In CFE module, we use the combination of multi-branch convolution and dilated convolution to achieve efficient extraction and multi-scale enhancement of small object features. The overall design of the module consists of three main branches, which undertake different feature processing tasks, and finally maximize the effectiveness of features by means of feature aggregation.

Firstly, the design focus of the first branch is to extract rich semantic information using a multi-branch convolutional structure and expand the receptive field in the process, so as to enhance the expression ability of context information. We performed a 1×1 convolution operation on the input feature mapping, initially adjusted the number of channels for subsequent processing, reduced the computational cost and laid a foundation for subsequent multi-branch processing. Then, the processed input features were input into three branch quasi-convolution operations, among which three branches contained a branch with only one convolution kernel as 3×3 standard convolution. Two branches consisting of a standard strip convolution with a size of 1×7 and 7×1 and a 3×3 dilated convolution with a dilated rate of 7, respectively, effectively capture the asymmetric and directional information in the features through the extended characteristics of the long axis receptive field of the strip convolution. Meanwhile, the dilated convolution expands the receptive field without increasing the number of parameters by introducing sparse receptive fields. In this way, the long distance dependence between the context information and the object is captured, and the features on the three branches are concat operation, the 1×1 convolution is input for processing. The features through the first main branch not only contain local details, but also retain global context information, and effectively improve the receptive field through the combination of strip convolution and dilated convolution. The multi-scale feature expression capability of the module for small objects is enhanced significantly. In addition, compared with the method of using large convolution kernel directly, the multi-branch design can significantly reduce the computational complexity and parameters while ensuring the effect of receptive field expansion, so as to realize the lightweight of the module. Second, the second branch is a residual structure composed of 1×1 convolution. The residual structure forms an equivalent mapping, and its main function is to directly retain input features through the equivalent mapping mechanism to avoid the loss of key features of small objects in multi-branch convolution operations. The introduction of residual structure not only ensures the integrity of the feature flow but also makes the CFE module better adapt to the feature representation requirements of different scale objects. Through this design, the fine-grained features of small objects are preserved, providing accurate scale information for subsequent feature fusion. Last, the third branch is the input of the original global feature information, which supplements the global information on the basis of local feature enhancement, so as to improve the network’s perception of the overall feature of the object. The retention of global features is particularly important for the detection of small objects, because the semantic information of small objects is sparse, and it is easy to be limited by local information. The introduction of global features can effectively improve the model’s context-aware ability of small objects, and further enhance the detection robustness. Finally, context information, key information, and global information are added element by element to retain and extract effective information of small objects to the maximum extent. This feature fusion mechanism realizes the effective integration of context information, key feature information and global information, and enhances the object features from three different scales. Specifically, the enhancement of context information significantly improves the semantic saturation of small objects, the retention of key feature information ensures the fine-grained description of small objects, and the addition of global information enhances the overall consistency of the object characteristics. The formulas processed by the MDFA module are expressed as follows: Equations 5–8:

(5) $$I_{1}=C_{3\times 3}\left[C_{1\times 1}\left(F\right)\right]$$

(6) $$I_{2}=DC_{r=7}^{3\times 3}\left\{C_{1\times 7}\left\{C_{7\times 1}\left[C_{1\times 1}\left(F\right)\right]\right\}\right\}$$

(7) $$I_{3}=DC_{r=7}^{3\times 3}\left\{C_{7\times 1}\;\left\{C_{1\times 7}\left[C_{1\times 1}\left(F\right)\right]\right\}\right\}$$

(8) $$F_{out}=Concat\left(I_{1},I_{2},I_{3}\right)⊕C_{1\times 1}\left(F\right)⊕F$$

Where $C_{1\times 1}\left(.\right)$, $C_{3\times 3}\left(.\right)$, $C_{1\times 7}\left(.\right)$, and $C_{7\times 1}\left(.\right)$ represent the standard convolution operations with kernel sizes of 1×1, 3×3, 1×7, and 7×1, respectively. $DC_{r=7}^{3\times 3}$ represents the $C_{3\times 3}\left(.\right)$ dilated convolution operation with an expansion rate of 7, Concat represents feature mapping concatenation operation, ⊕ represents Element-wise Addition, F is the input feature, $I_{1},I_{2},I_{3}$ represent the output feature map of the first three branches after standard convolution and dilated convolution, *F_out_* is the output feature.

### 3.4 Extra detect head

During feature extraction, the object detection model sends the feature images P3, P4, and P5 of three different resolutions obtained by the backbone network into the neck for feature fusion. This is because with the superposition of downsampling or convolution operations, the receptive field gradually expands, and the high-level feature map can capture richer semantic information, which is sufficient for the object detection of general objects. However, for a large number of small objects to be detected, due to little information in small objects, their size, location, and other feature information may be gradually lost with the increasing number of model layers, which is not conducive to accurate object recognition and positioning (Xu et al., 2022), and the prediction head cannot obtain enough feature information from the feature map, resulting in low recognition accuracy. Shallow feature maps have smaller receptive fields, pay more attention to detail information, and have higher spatial resolution and accurate location information, which is suitable for small object detection tasks that lack feature information and are difficult to pinpoint.

In order to retain more shallow features and small object location information, feature map P2 with the highest resolution is introduced. By reducing downsampling times and retaining more detailed information, feature map P2 extracted through the backbone network is fused with feature maps of other scales to improve the richness of fusion features. Moreover, an additional P2 feature small object prediction head is constructed by using the fused features, so that the model has more location information and feature information of the small object, effectively reducing the location feature loss during feature downsampling, enhancing the context information of the small object, and improving the location detection accuracy of the small object. And combine with the other three prediction heads, it can well mitigate the negative effects caused by drastic changes in object scale.

### 3.5 MDFE-Net

**Figure 4:**
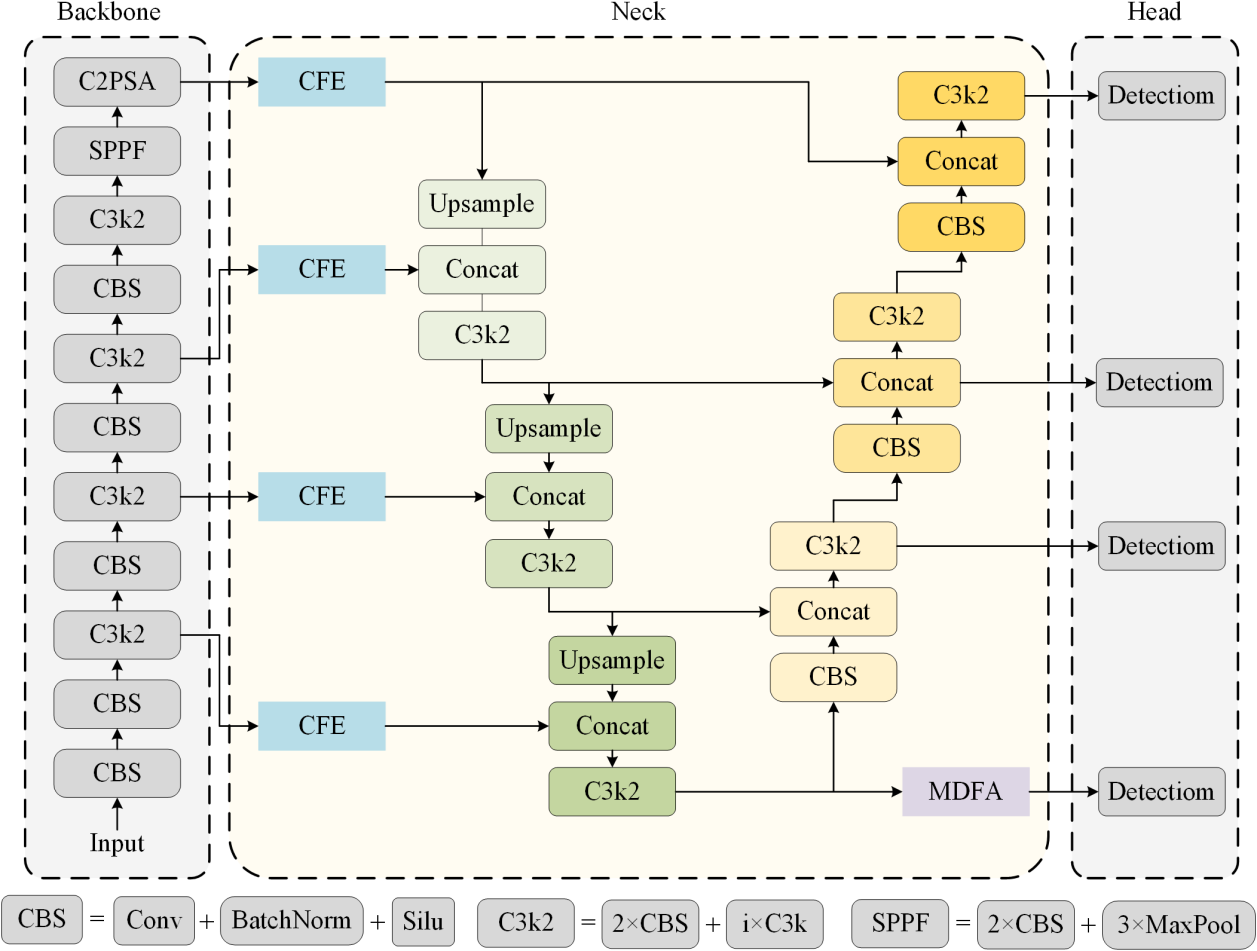
Overall structure of the proposed MDFE-Net, which includes baseline model components (CBS, C3k2, and SPPF).

To effectively address small object detection challenges, the proposed innovative modules MDFA and CFE are introduced into the object detection model YOLO11N of YOLO series methods, and the lightweight version model YOLO11N is used as the benchmark network framework. An innovative model multiscale dilate feature enhancement network (MDFE-Net) was constructed, and the overall framework of MDFE-Net was shown in **Figure 4**. CSPDarkNet53 (Mahasin and Dewi, 2022) is employed as the backbone network to efficiently extract hierarchical multi-scale features from images. The neck structure is used for feature fusion, combine with multi-resolution feature maps to improve the awareness of small object context information. The detection head structure is used to classify and locate the object. The CFE module is used to enhance the image context feature information of four different resolutions output by the trunk to improve the feature extraction ability for small objects. Meanwhile, in order to make better use of the details of the underlying features, the MDFA module is used to carry out multi-scale feature aggregation for the underlying features to enhance the attentional expression ability of the underlying features. To further enhance the detection accuracy of the location of small objects, we introduce the underlying feature diagram P2 into the detection head and build additional detection head based on this, which effectively improves the classification and location performance of the model for small objects.

## 4 Experiments

### 4.1 Experimental setup

#### 4.1.1 Experimental dataset description

We chose three different types of small object datasets, including two public datasets and one self-built dataset.

**Figure 5:**
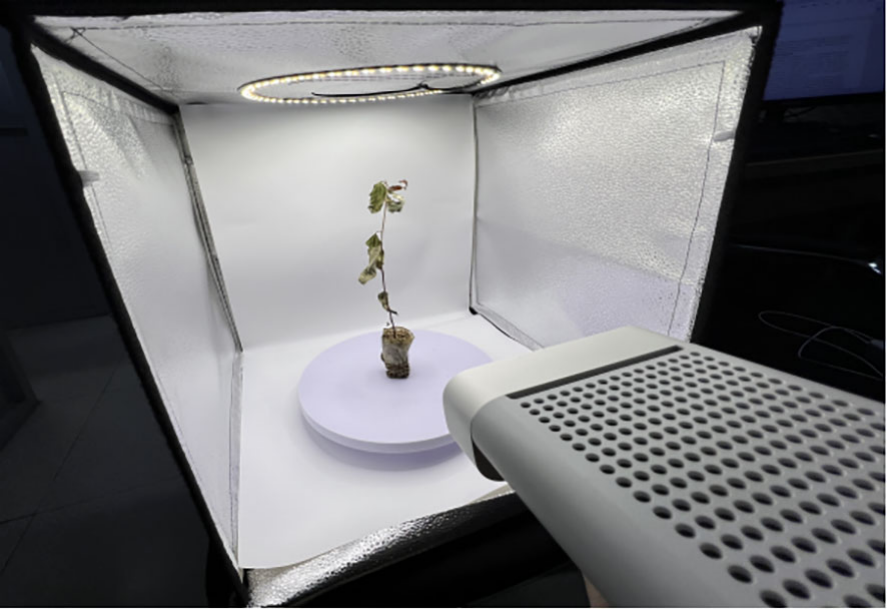
Self-built image collection room for plant seedlings.

1. VisDrone (Cao et al., 2021): It is a large-scale UAV view dataset in a realistic scene, which contains a large number of small objects, diverse data distribution, and complex detection scenes, which makes the dataset more challenging. The dataset contains 10,209 still images from drones in different areas of 14 cities, covering 10 common object categories in traffic scenarios, including about 540,000 instances.
2. GTSDB (Zhang et al., 2020): The German Traffic Sign Detection Benchmark dataset is a traffic sign detection benchmark dataset in Germany, which contains a total of 900 images of 1360×800 pixels and 4 categories of label types, and has a large number of small traffic signs.
3. PSD-Node: Plant Seedling Dataset-Node is a dataset for plant seedling node detection, which is collected and labeled by us in an independent seedling image data collection room (as shown in **Figure 5**. There are 1350 original seedling images collected in total, including 810 training sets. There are 270 verification sets and 270 test sets, which contain tens of thousands of small object labels for seedling nodes. The dataset has the following characteristics: (1) The labels of seedling nodes in PSD-Node belong to small objects in the definition of relative size. (2) The goal of a large number of seed and seedling nodes in PSD-Node is to more effectively verify the model’s performance in detecting small objects under low light conditions and under the condition of blade occlusion. (3) PSD-Node belongs to the small object dataset in the agricultural field, which has reference value for improving the small object detection performance of the model in the agricultural field.

#### 4.1.2 Evaluation metrics

We used P (Precision), R (Recall), F1-score, AP (averaged over IoU thresholds from 0.50 to 0.95 with a step of 0.05), and AP50 (average precision at IoU = 0.50) as the main evaluation metrics for the model. In addition to AP and AP50, we report scale-aware metrics APs, APm, and APl to better evaluate performance across different object sizes. These metrics follow the common small, medium, and large scale partition used in standard detection evaluation. In addition, we report GFLOPs and parameter count in **Tables 1**, **2** to provide an efficiency-related reference under the same input resolution.

**Table 1:** Comparisons of MDFE-Net with state-of-the-art algorithms in VisDrone.

Model	P	R	F1	AP50	AP	APs	APm	APl	GFLOPs	Parameter
YOLOv5N(2020)	0.379	0.294	0.331	0.266	0.152	0.046	0.154	0.231	7.1	2.18M
YOLOv6N(2022)	0.381	0.289	0.329	0.268	0.148	0.051	0.210	0.316	11.4	4.15M
YOLOv8N(2023)	0.376	0.288	0.326	0.264	0.147	0.059	0.225	0.339	8.1	3.00M
YOLOv9T(2024)	0.385	0.291	0.331	0.270	0.154	0.059	0.221	0.341	10.7	2.00M
YOLOv10N(2024)	0.383	0.292	0.331	0.268	0.149	0.063	0.224	0.292	10.8	2.69M
YOLOv11N(2024)	0.380	0.293	0.331	0.267	0.149	0.058	0.225	0.316	6.3	2.58M
RT-DETR-L(2024)	0.384	0.285	0.327	0.278	0.150	/	/	/	108.0	32.81M
Hyper-YOLO-N(2025)	0.402	0.314	0.353	0.292	0.161	0.066	0.240	0.348	10.8	3.94M
YOLOv12N(2025)	0.395	0.299	0.340	0.275	0.156	0.057	0.224	0.346	6.3	2.55M
FBRT-YOLO-N(2025)	0.393	0.300	0.340	0.277	0.158	0.063	0.230	0.310	6.7	0.85M
EDSOD(2025)	0.245	0.437	0.314	0.245	0.135	0.073	0.197	0.291	88.1	23.45M
MDFE-Net(Ours)	**0.410**	**0.326**	**0.363**	**0.304**	**0.172**	**0.077**	**0.268**	**0.361**	13.6	3.22M

The bolded values represent the evaluation metric with the best performance after comparing all the models.

**Table 2:** Comparisons of MDFE-Net with state-of-the-art algorithms in GTSDB.

Model	P	R	F1	AP50	AP	APs	APm	APl	GFLOPs	Parameter
YOLOv5N(2020)	0.953	0.833	0.889	0.925	0.748	0.523	0.783	0.895	7.1	2.18M
YOLOv6N(2022)	0.914	0.854	0.883	0.911	0.728	0.501	0.773	0.879	11.4	4.15M
YOLOv8N(2023)	0.913	0.887	0.900	0.921	0.758	0.534	0.766	0.838	8.1	3.00M
YOLOv9T(2024)	0.923	0.846	0.883	0.905	0.744	0.543	0.788	0.931	10.7	2.00M
YOLOv10N(2024)	0.956	0.846	0.898	0.929	0.767	0.510	0.786	0.883	10.8	2.69M
YOLOv11N(2024)	0.953	0.859	0.905	0.926	0.773	0.541	0.789	0.891	6.3	2.58M
RT-DETR-L(2024)	0.960	0.874	0.915	0.935	0.778	/	/	/	108.0	32.81M
Hyper-YOLO-N(2025)	0.911	0.896	0.903	0.939	0.769	0.515	0.787	0.887	10.8	3.94M
YOLOv12N(2025)	0.958	0.816	0.881	0.921	0.744	0.491	0.812	0.878	6.3	2.55M
FBRT-YOLO-N(2025)	0.961	0.884	0.921	0.933	0.769	0.577	0.817	0.855	6.7	0.85M
EDSOD(2025)	0.825	**0.919**	0.869	0.825	0.658	0.436	0.762	0.774	88.1	23.45M
MDFE-Net(Ours)	**0.964**	0.898	**0.930**	**0.952**	**0.805**	**0.593**	**0.828**	**0.910**	13.6	3.22M

The bolded values represent the evaluation metric with the best performance after comparing all the models.

#### 4.1.3 Other details

We trained on 1 GPU (NVIDIA GeForce RTX 2080), Intel(R)Core(TM)i7–8700 CPU, and Windows 10 operating system, and selected the best performance as the experimental results. We selected Stochastic Gradient Descent (SGD) as the network optimizer. The epochs and batch size were set to 300 and 4, respectively. In the training process, the initial learning rate was set to 0.01, and the cosine annealing strategy was used to reduce the learning rate. Momentum was set to 0.937 and weight loss was set to 0.0005. To ensure a fair comparison, we excluded the use of pre-training and self-distillation strategies for all methods used for comparison, and in addition, recognizing the potential impact of input image size on evaluation, we normalized the input resolution for all data images to 640×640, a common choice in the field of object detection. For reproducibility, we additionally report the main software environment: Python 3.8, PyTorch 2.0.1, CUDA 11.7, and cuDNN 8.5.0. Unless otherwise specified, we fix the random seed (e.g., 42) for Python, NumPy, and PyTorch, and enable deterministic settings where applicable.

Training objective and loss: To avoid ambiguity, MDFE-Net follows the same training objective as the YOLO11N baseline. Specifically, we keep the default YOLO11N loss formulation (classification, localization, and objectness terms) and their weights unchanged. Moreover, the label assignment strategy is identical to the baseline, and we do not introduce any additional loss terms, customized matching rules, or auxiliary supervision. Therefore, the performance gains mainly come from the proposed MDFA and CFE modules rather than changes in the training objective.

### 4.2 Comparisons with state-of-the-art algorithms

To evaluate MDFE-Net, we selected the current advanced single-stage object detection methods for comparative experiments, including YOLOv5N (Jocher, 2020), YOLOv6N (Li et al., 2023a), YOLOv8N (Jocher et al., 2023), YOLOv9T (Wang et al., 2024b), YOLOv10N (Wang et al., 2024a), YOLO11N (Khanam and Hussain, 2024), Hyper-YOLO-N (Feng et al., 2024), and YOLOv12N (Tian et al., 2025). At the same time, RT-DETR-L (Zhao et al., 2024) based on the end-to-end non-CNN framework was selected for comparative experiments.

**Figure 6:**
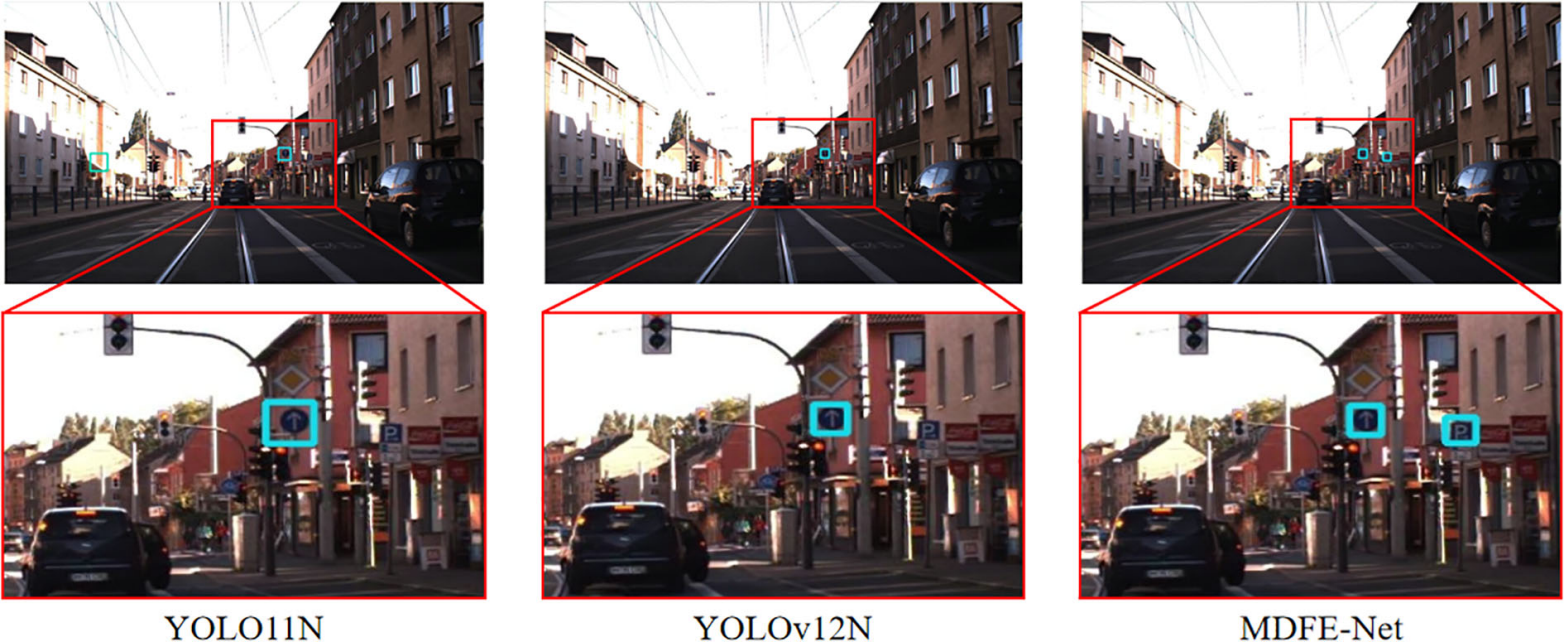
Visualization comparison of YOLO11N, YOLOv12N, and MDFE-Net on the PSD-Node dataset. The bottom row shows a zoomed-in region. Red bounding boxes denote false positives. YOLO11N and YOLOv12N produce an additional false alarm in the enlarged area, whereas MDFE-Net suppresses this false detection.

1. PSD-Node: **Table 3** shows that MDFE-Net achieves the best performance on the PSD-Node dataset in terms of F1, AP50, and AP. Compared with the baseline YOLO11N, MDFE-Net improves F1, AP50, and AP by 3.7%, 4.8%, and 6.8%, respectively. Compared with the recent SOTA Hyper-YOLO-N, MDFE-Net still achieves consistent gains of 1.5%, 1.4%, and 1.4% in F1, AP50, and AP, respectively. Specifically, MDFE-Net achieves 84.8% F1, 89.5% AP50, and 47.6% AP. Although MDFE-Net attains slightly lower precision than FBRT-YOLO-N, it remains the second-highest among all compared methods, while achieving the best results on F1, AP50, and AP. Moreover, MDFE-Net also outperforms other strong detectors included in **Table 3**, such as RT-DETR-L, YOLOv12N, and FBRT-YOLO-N, demonstrating the effectiveness of our approach on this challenging dataset. We provide visual examples in **Figure 6** to compare MDFE-Net with the advanced detectors YOLO11N and YOLOv12N. As shown in the zoomed-in region, YOLO11N and YOLOv12N produce an additional false positive (highlighted in red), whereas MDFE-Net suppresses this false alarm, indicating improved robustness and accuracy for small-object detection.

**Table 3:** Comparisons of MDFE-Net with state-of-the-art algorithms in PSD-Node.

Model	P	R	F1	AP50	AP	Parameter
YOLOv5N(2020)	0.858	0.788	0.822	0.863	0.429	2.18M
YOLOv6N(2022)	0.865	0.776	0.818	0.844	0.394	4.15M
YOLOv8N(2023)	0.855	0.775	0.813	0.847	0.395	3.00M
YOLOv9T(2024)	0.863	0.764	0.810	0.835	0.382	2.00M
YOLOv10N(2024)	0.853	0.771	0.810	0.856	0.419	2.69M
YOLOv11N(2024)	0.860	0.768	0.811	0.847	0.408	2.58M
RT-DETR-L(2024)	0.849	0.798	0.823	0.865	0.437	32.81M
Hyper-YOLO-N(2025)	0.863	0.805	0.833	0.881	0.462	3.94M
YOLOv12N(2025)	0.868	0.791	0.828	0.866	0.434	2.55M
FBRT-YOLO-N(2025)	**0.890**	0.779	0.831	0.875	0.445	0.85M
MDFE-Net(Ours)	0.884	**0.815**	**0.848**	**0.895**	**0.476**	3.22M

The bolded values represent the evaluation metric with the best performance after comparing all the models.

**Figure 7:**
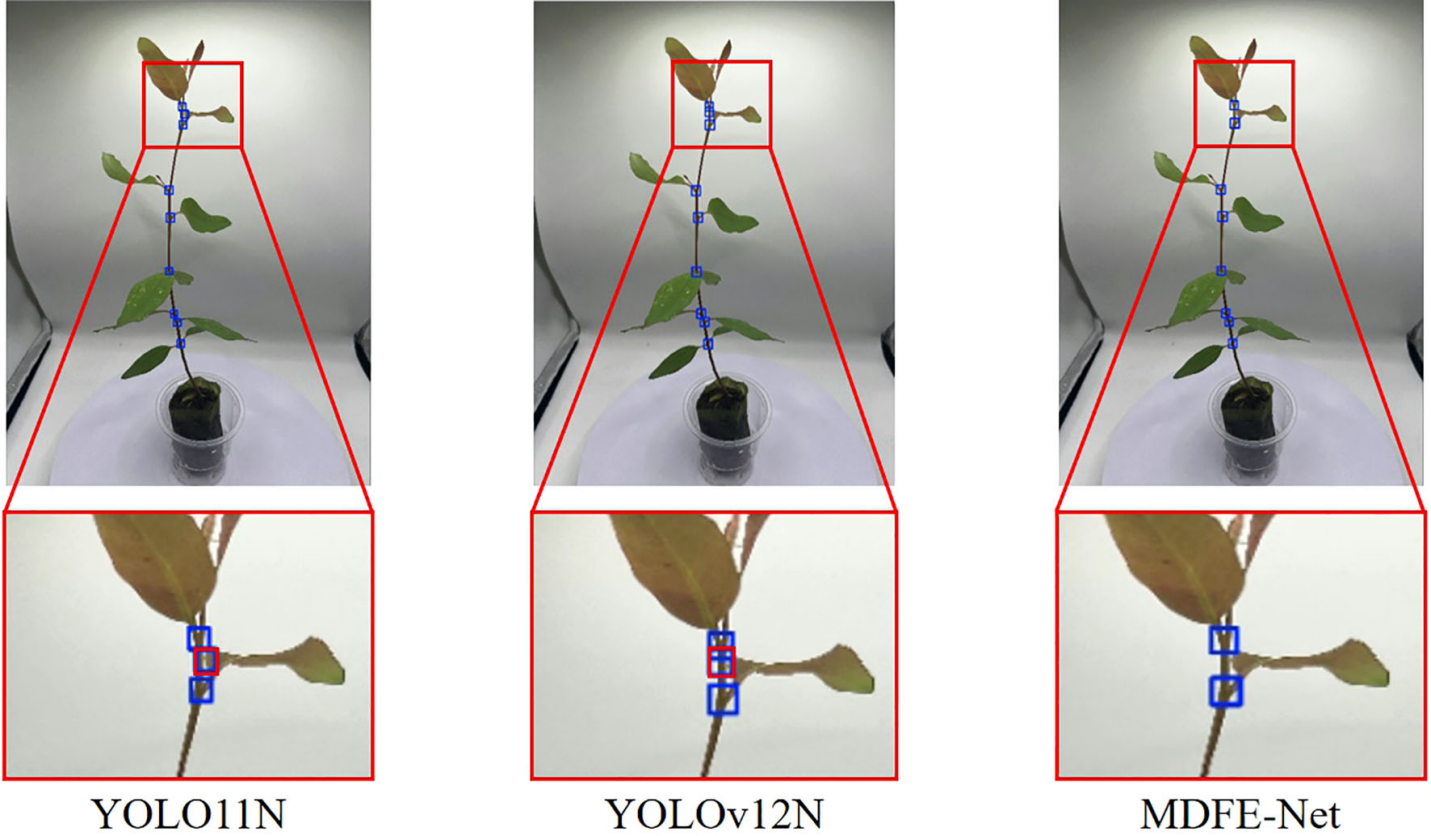
Qualitative comparison of YOLO11N, YOLOv12N, and MDFE-Net on VisDrone. Purple boxes indicate motorcycles, cyan boxes indicate cars, and blue boxes indicate vans. In this dense small-object scene, MDFE-Net detects more valid instances with fewer missed detections in the zoomed-in region compared with YOLO11N and YOLOv12N.

**Figure 8:**
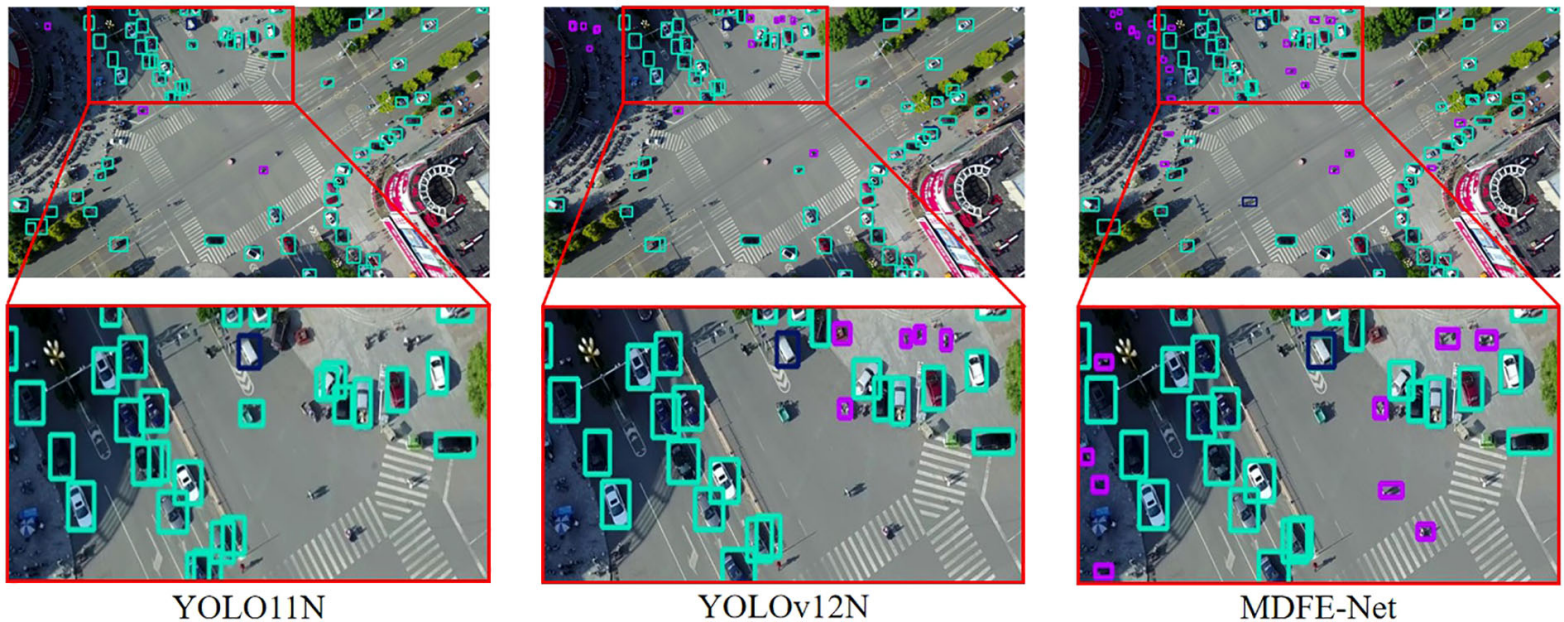
Comparison of visualization results of YOLO11N, YOLOv12N, and MDFE-Net on GTSDB. Compared with the advanced YOLO11N and YOLOv12N, MDFE-Net has better detection performance for small objects in images.

2. VisDrone: **Table 1** shows that MDFE-Net outperforms the baseline YOLO11N on the VisDrone dataset, improving F1, AP50, and AP by 3.2%, 3.7%, and 2.3%, respectively. In addition, MDFE-Net achieves better scale-aware performance with APs/APm/APl of 0.077/0.268/0.361, showing clear gains across different object scales, especially for small objects. Compared with recent state-of-the-art lightweight detectors included in **Table 1**, MDFE-Net achieves the best overall performance under the same evaluation setting. We also report GFLOPs (computed at 640×640 input) together with parameter count to characterize computational cost and provide a more complete view of accuracy-efficiency trade-offs. To further illustrate the effectiveness of MDFE-Net on small-object detection, we provide a qualitative comparison in **Figure 7** against two strong baselines, YOLO11N and YOLOv12N, on the VisDrone dataset. Different categories are indicated by different colors: purple denotes motorcycles, cyan denotes cars, and blue denotes vans. In this challenging scene with dense small objects, YOLO11N and YOLOv12N exhibit more missed detections, particularly for motorcycles, while MDFE-Net detects more valid instances with fewer misses in the zoomed-in region. These observations are consistent with the quantitative results in **Table 1**, where MDFE-Net achieves the best overall performance on VisDrone.

3. GTSDB: **Table 2** shows that MDFE-Net achieves consistent improvements on the GTSDB dataset. Compared with the baseline YOLO11N, MDFE-Net improves F1, AP50, and AP by 2.5%, 2.6%, and 3.2%, respectively. Moreover, MDFE-Net obtains APs/APm/APl of 0.593/0.828/0.910, demonstrating strong performance across different object scales. We further report GFLOPs (computed at 640 ×640 input) and parameter count to provide an efficiency-related reference under the same evaluation setting. Although the recall of MDFE-Net is slightly lower than that of EDSOD, it remains the second-highest among all compared methods, while MDFE-Net achieves the best results on the other major metrics (F1, AP50, AP, and APs/APm/APl), indicating strong overall detection performance. We further validate the effectiveness of our proposed method in small object detection tasks by providing an example of visualization of the GTSDB dataset in **Figure 8**, comparing MDFE-Net with the state-of-the-art object detectors YOLO11N and YOLOv12N. It is not difficult to see that the proposed method can effectively improve the accuracy of traffic small object detection model, and has better small object detection performance and lower miss rate than other SOTA models.

### 4.3 Ablation studies

As for the MDFA module, the original design intention of the module was to extract more underlying feature information in a larger range of receptive fields to improve the detection performance of small objects through the characteristics of multi-scale cavity convolution, so we placed the module in four feature output paths in YOLO11N for ablation study to verify the placement position of the module in the network. According to **Table 4** the experimental results show that the P2 feature layer is the optimal location for MDFA module. The main reason is that the P2 feature layer, as the bottom feature output of the network, has the highest feature map resolution and contains the richest detail information and texture data, which is crucial for the detection of small objects. Through the design of multi-scale cavity convolution, MDFA module can extract global context information from a wider range of receptive fields while maintaining the integrity of high-resolution feature maps, and strengthen the underlying features by combining multi-scale feature aggregation mechanisms. This design enables the module to give full play to the advantages of rich detail information of the P2 layer, and provides more accurate feature support for the classification and positioning of small objects. In contrast, when MDFA modules are placed in mid-to-high level feature output paths (such as P3, P4, or P5), although these layers contain stronger semantic information, due to their lower resolution and sparse object details, it is difficult to fully utilize the characteristics of the MDFA module. At the same time, high-level features pay more attention to the semantic expression of large objects, and there is a certain deviation between the features and the requirements of small object detection tasks, so it will cause interference with small object detection. Therefore, adding MDFA modules to layer P3, P4, or P5, or adding MDFA modules to all four layers, is not as significant as adding MDFA modules to layer P2.

**Table 4:** Ablation study of MDFA module.

P5	P4	P3	P2	P	R	F1	AP50	AP
✔				0.863	0.783	0.821	0.872	0.444
	✔			0.879	0.783	0.828	0.880	0.458
		✔		0.859	0.815	0.836	0.883	0.449
			✔	0.884	0.815	0.848	0.895	0.476
✔	✔	✔	✔	0.882	0.766	0.820	0.870	0.453

To verify the validity of our proposed method, we conducted ablation study on the PSD-Node validation set and analyzed the impact of introducing various modules into the baseline network, as shown in **Table 5**.

**Table 5:** Ablation study of each module in PSD-Node EDH denoted as extra detect head.

EDH	MDFA	CFE	P	R	F1	AP50	AP
			0.860	0.768	0.811	0.847	0.408
	✔		0.855	0.801	0.827	0.869	0.446
		✔	0.860	0.805	0.832	0.873	0.450
	✔	✔	0.864	0.808	0.835	0.875	0.452
✔			0.870	0.796	0.831	0.874	0.450
✔	✔		0.879	0.808	0.842	0.884	0.458
✔		✔	0.879	0.798	0.837	0.890	0.471
✔	✔	✔	0.884	0.815	0.848	0.895	0.476

Firstly, the introduction of MDFA modules can significantly improve performance. It can effectively utilize low-level feature information through a multi-scale attention mechanism, improve the localization and detection ability of smaller objects, and better capture multi-scale local feature information. The introduction of the MDFA module alone resulted in a 1.6% increase in F1, a 2.2% increase in AP50, and a 3.8% increase in AP. Second, the introduction of the CFE module alone can extract a variety of feature semantic information through the multi-branch convolution structure, and dilated convolution can be used to increase the receptive field and obtain richer local context information. With the addition of the CFE module, F1, AP50, and AP improved by 2.1%, 2.6%, and 4.2%, respectively, due to the ability to extract rich contextual information through the multi-branch convolutional structure. The separate introduction of MDFA and CFE modules can be significantly improved. When the above two modules are introduced together, finally, F1, AP50, and AP increase by 2.4%, 2.8%, and 4.6% respectively, indicating that the combination of MDFA and CFE modules can produce better results than the separate modules. Finally, low-level feature maps typically contain richer spatial and local details, essential for accurate localization. We introduce the feature map P2 with the highest resolution. By reducing the downsampling times and retaining more detailed information, P2 is fused with feature maps of other scales to increase the sensitivity of small objects, and the detection head is used to output positioning and classification. It can be seen that F1, AP50, and AP increase by 2.0%, 2.7%, and 4.2% respectively after the feature map of layer P2 is introduced and the detection head is added. We combine MDFA and CFE modules with P2 layer detect head into network. F1, AP50, and AP increased by 3.7%, 4.8%, and 6.8% respectively, which made the model achieve the best detection effect, and also proved the effectiveness of our proposed MDFA and CFE innovative modules.

## 5 Conclusion

In this paper, we proposed two innovative plug-and-play modules, the MDFA and CFE modules, tailored specifically for small object detection, and integrated them into the YOLO11N framework to construct an effective small-object detector. We name this module the multi-scale dilated feature aggregation (MDFA) module. The proposed MDFA module improves multi-scale context modeling via dilated convolutions, thereby enriching the feature information of small objects. In addition, the proposed Context Feature Enhancement (CFE) module improves local feature perception and preserves informative cues for small objects to the greatest extent. It applies dilated convolution to increase the receptive field and enrich the context information, so as to better solve the detection problem of small objects. Finally, we demonstrate through experimental results that MDFE-Net achieves state-of-the-art performance over the current SOTA model in terms of small objects. In future work, we plan to further improve robustness under challenging conditions such as heavy occlusion and background clutter in dense small-object scenes. We also plan to strengthen fine-grained category discrimination for confusing classes. In addition, we will explore deployment-oriented optimization and validation on more domains to enhance generalization.

## Data Availability

The original contributions presented in the study are included in the article/supplementary material. Further inquiries can be directed to the corresponding authors.
